# See, Hear, or Feel – to Speak: A Versatile Multiple-Choice Functional Near-Infrared Spectroscopy-Brain-Computer Interface Feasible With Visual, Auditory, or Tactile Instructions

**DOI:** 10.3389/fnhum.2021.784522

**Published:** 2021-11-25

**Authors:** Laurien Nagels-Coune, Lars Riecke, Amaia Benitez-Andonegui, Simona Klinkhammer, Rainer Goebel, Peter De Weerd, Michael Lührs, Bettina Sorger

**Affiliations:** ^1^Department of Cognitive Neuroscience, Faculty of Psychology and Neuroscience, Maastricht University, Maastricht, Netherlands; ^2^Maastricht Brain Imaging Center, Maastricht, Netherlands; ^3^Zorggroep Sint-Kamillus, Bierbeek, Belgium; ^4^MEG Core Facility, National Institutes of Mental Health, Bethesda, MD, United States; ^5^Department of Psychiatry and Neuropsychology, Faculty of Health Medicine and Life Sciences, Maastricht University, Maastricht, Netherlands; ^6^School for Mental Health and Neuroscience, Maastricht University, Maastricht, Netherlands; ^7^Brain Innovation B.V., Maastricht, Netherlands; ^8^Maastricht Centre for Systems Biology, Maastricht University, Maastricht, Netherlands

**Keywords:** functional near-infrared spectroscopy (fNIRS), brain-computer interface (BCI), motor imagery (MI), mental drawing, sensory encoding modality, four-choice communication, temporal encoding, reliability over time

## Abstract

Severely motor-disabled patients, such as those suffering from the so-called “locked-in” syndrome, cannot communicate naturally. They may benefit from brain-computer interfaces (BCIs) exploiting brain signals for communication and therewith circumventing the muscular system. One BCI technique that has gained attention recently is functional near-infrared spectroscopy (fNIRS). Typically, fNIRS-based BCIs allow for brain-based communication via voluntarily modulation of brain activity through mental task performance guided by visual or auditory instructions. While the development of fNIRS-BCIs has made great progress, the reliability of fNIRS-BCIs across time and environments has rarely been assessed. In the present fNIRS-BCI study, we tested six healthy participants across three consecutive days using a straightforward four-choice fNIRS-BCI communication paradigm that allows answer encoding based on instructions using various sensory modalities. To encode an answer, participants performed a motor imagery task (mental drawing) in one out of four time periods. Answer encoding was guided by either the visual, auditory, or tactile sensory modality. Two participants were tested outside the laboratory in a cafeteria. Answers were decoded from the time course of the most-informative fNIRS channel-by-chromophore combination. Across the three testing days, we obtained mean single- and multi-trial (joint analysis of four consecutive trials) accuracies of 62.5 and 85.19%, respectively. Obtained multi-trial accuracies were 86.11% for visual, 80.56% for auditory, and 88.89% for tactile sensory encoding. The two participants that used the fNIRS-BCI in a cafeteria obtained the best single- (72.22 and 77.78%) and multi-trial accuracies (100 and 94.44%). Communication was reliable over the three recording sessions with multi-trial accuracies of 86.11% on day 1, 86.11% on day 2, and 83.33% on day 3. To gauge the trade-off between number of optodes and decoding accuracy, averaging across two and three promising fNIRS channels was compared to the one-channel approach. Multi-trial accuracy increased from 85.19% (one-channel approach) to 91.67% (two-/three-channel approach). In sum, the presented fNIRS-BCI yielded robust decoding results using three alternative sensory encoding modalities. Further, fNIRS-BCI communication was stable over the course of three consecutive days, even in a natural (social) environment. Therewith, the developed fNIRS-BCI demonstrated high flexibility, reliability and robustness, crucial requirements for future clinical applicability.

## Introduction

The motor system plays a pivotal role in natural human communication. Any disruption to this system can negatively affect our ability to communicate. Severely motor-disabled patients lose the ability to communicate in an intuitive manner. For example, patients suffering from the “locked-in” syndrome (Plum and Posner, 1982) are aware but have lost the ability to speak. Patients with the “classic” locked-in syndrome and those in early stages of amyotrophic lateral sclerosis (ALS), can use eye movement for basic communication. However, some patients suffer from deficits in the oculomotor system, such as those with “complete” locked-in syndrome (CLIS) or in late stages of ALS. These patients are particularly in need of motor-independent communication means that rely on central nervous system activation. Restoring basic communication in these patients can have a large impact on their quality of life (Kübler et al., 2005; Rousseau et al., 2012).

Brain-computer interfacing (BCI) enables motor-independent communication through brain-based encoding of intention. The BCI user willfully modifies her/his brain activation, which is recorded using functional neuroimaging and from which an answer is decoded. The most widely used imaging method in the context of BCI is the electroencephalogram (EEG), which records neuro-electric brain signals (Kubler et al., 2009; Marchetti et al., 2013; Lazarou et al., 2018; Won et al., 2019).

However, not everyone can control an EEG-based BCI (Allison and Neuper, 2010). Especially in the late stages of ALS, when patients lose all ocular control and enter a CLIS state, interpretable visual signals are rare (Borgheai et al., 2020). This highlights the need for alternatives for the heterogeneous population of patients who have to rely on a BCI. In this context, hemodynamic responses, relying on blood flow instead of electric signals, constitute a viable alternative. Successful communication has been demonstrated using functional magnetic resonance imaging (fMRI) paradigms in healthy participants (Sorger et al., 2009, 2012) and patients (Monti et al., 2010) when using two or three mental imagery tasks. Nevertheless, fMRI has its drawbacks, such as high costs, immobility and participant-specific contra-indications to being in a strong magnetic field (Irani et al., 2007; Naci et al., 2012; Scarapicchia et al., 2017). There is a need for these promising hemodynamic paradigms to be transferred to a portable neuroimaging method that can be used in ecologically valid environments in which communication typically takes place (Sorger et al., 2009, 2012; Naci et al., 2012).

Functional near-infrared spectroscopy (fNIRS) is such an alternative method. It being portable, relatively affordable and easier to operate than fMRI (Naci et al., 2012). This neuroimaging method measures the hemodynamic response using near-infrared light emitters and sensors, called optodes. The term “channel” is used to define a specific optode pair (one emitter and one receiver optode). Cortical activity can be detected through relative concentration changes in oxygenated (HbO) and de-oxygenated (HbR) hemoglobin. Validation studies have shown that fNIRS signals correlate strongly with fMRI signals (Huppert et al., 2006; Cui et al., 2011; Scarapicchia et al., 2017), despite lower spatial resolution and signal-to-noise ratio of fNIRS.

In recent years, methodological advances in fNIRS hardware and signal processing have resulted in a steady increase of fNIRS publications (Naseer and Hong, 2015b; Pinti et al., 2018b). Similar to the fMRI paradigms, most fNIRS-BCI research has focused on binary communication via mental imagery tasks (Naito et al., 2007; Nagels-Coune et al., 2017, 2020; Abdalmalak et al., 2020), with a few studies showing effective decoding of four (Batula et al., 2014; Naseer and Hong, 2015a), or six (Benitez-Andonegui et al., 2020) answer options. Mental imagery is typically guided with a single sensory encoding modality, mainly visual or auditory. Answer decoding is often done using multivariate classification techniques that rely on spatial features of the different mental imagery tasks (Batula et al., 2014; Naseer and Hong, 2015b; Weyand and Chau, 2015; Hong et al., 2018). Despite the technological and methodological advances, most fNIRS-BCI studies so far have been limited to laboratory environments (Naseer and Hong, 2015b). FNIRS-BCIs have only been tested in a handful of patient studies (Naito et al., 2007; Abdalmalak et al., 2017b; Borgheai et al., 2020). For an fNIRS-BCI to reach end-users, its setup should be straightforward and flexible both in terms answer encoding and decoding. Crucially, an fNIRS-BCI should also work reliably over time and in different environments.

**FIGURE 1:**
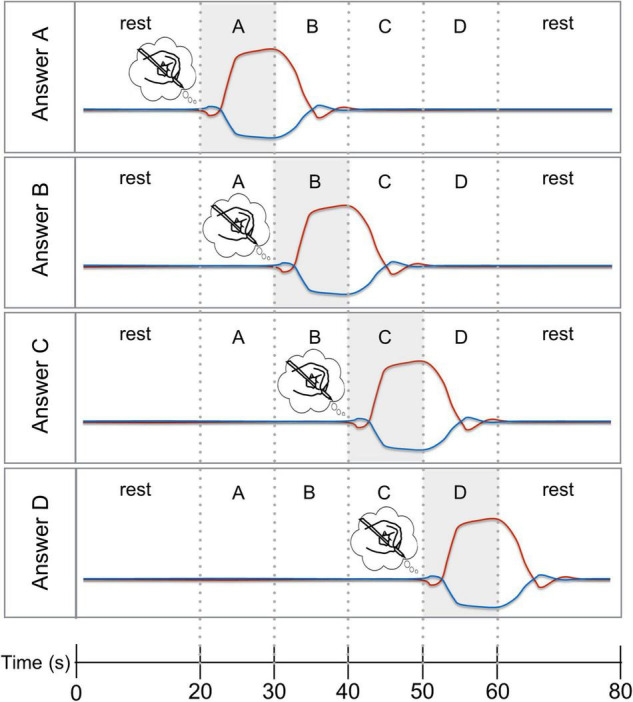
Four-choice temporal encoding paradigm with expected time courses. Hypothesized HbO (red line) and HbR (blue line) responses for the four answer options “A”, “B”, “C”, and “D”. To chose answer option “A” (top panel), the participant had to start mental imagery upon the cue presented 20 s after trial onset and stop the mental imagery when answer option “B” was presented. For the remaining trial time, the participant had to rest and await the subsequent encoding trial. The other three panels show the hypothesized HbO and HbR response for answer options “B”, “C”, and “D”.

Previous work from our lab has shown the potential of the temporal encoding paradigm (see Figure 1) in which answer options are presented in a serial manner (Benitez-Andonegui et al., 2020; Nagels-Coune et al., 2020). Thereby, participants can perform a single mental imagery task when their chosen answer option is presented. Other fNIRS studies have used paradigms that strongly rely on spatial discrimination of brain-activation patterns evoked by different mental imagery tasks (Batula et al., 2014; Naseer and Hong, 2015a; Weyand and Chau, 2015; Hong et al., 2018). The advantage of the temporal paradigm lies in its simplicity, which is enabled by the limited pre-training time it requires from the BCI user and the combined use of a single motor imagery task, (usually) a single fNIRS channel, and relatively basic univariate data analysis. Our lab has tested the temporal encoding paradigm using a two- (Nagels-Coune et al., 2020) and six-choice (Benitez-Andonegui et al., 2020) paradigm. In the current study, we aim to replicate the success of the temporal encoding paradigm using a four-choice paradigm. So far only two studies have tested a four-choice fNIRS-BCI in healthy participants. In a preliminary study by Batula et al. (2014), three participants used four motor imagery tasks, specifically right hand, left hand, right foot and left foot tapping, to communicate their answer. Data from the 18 fNIRS optodes was analyzed with a support vector machine, resulting in a mean single-trial accuracy of 45.7%. Naseer and Hong (2015a) asked ten healthy participants to use four distinct mental imagery tasks, namely right-hand motor imagery, left-hand motor imagery, mental arithmetic and mental counting, to encode four answer options. Using 32 fNIRS optodes and linear discriminant analysis to discern differentiable spatial patterns, a mean single-trial accuracy of 73.3% was reached.

Next to a convenient fNIRS-BCI paradigm, there is a need for flexibility in term of the sensory modality that guides the user to encode an answer option. Many EEG-based BCIs have focused on the visual modality (Allison and Neuper, 2010; Brunner et al., 2010; Treder and Blankertz, 2010). However, vision is one of the most affected senses in patients in need of a BCI (Gill-Thwaites and Munday, 2004; Riccio et al., 2012; Rousseau et al., 2012). Therefore, the auditory encoding modality has been explored in EEG-based BCIs (Kubler et al., 2009; Simon et al., 2015; Sugi et al., 2018) and fMRI-based BCIs (Monti et al., 2010; Naci and Owen, 2013). Encoding displays in the tactile modality remain relatively unexplored, with only a few EEG-based BCIs reported (Muller-Putz et al., 2006; Kaufmann et al., 2013; Lugo et al., 2014; Guger et al., 2017). However, tactile encoding might provide a critical solution for patients who are unable to use either visual or auditory BCI paradigms. Kaufmann et al. (2013) reported a LIS patient in whom the tactile modality was of superior benefit compared to the visual and auditory modalities in the context of an EEG-based BCI. To our knowledge, no study has yet explored the tactile encoding modality in the context of an fNIRS-BCI. Moreover, no BCI study has systematically explored three different sensory encoding modalities within the same participants employing an identical BCI paradigm.

Another critical factor for end-users is the reliability of the fNIRS-BCI. Most fNIRS-BCI studies were performed in a single session (Naito et al., 2007; Naseer and Hong, 2015a; Abdalmalak et al., 2017b; Nagels-Coune et al., 2017, 2020; Benitez-Andonegui et al., 2020), with the exception of the recent single-case study by Borgheai et al. (2020). Test-retest reliability of fNIRS signals has been assessed in non-BCI fNIRS research (Plichta et al., 2006; Blasi et al., 2014; Wiggins et al., 2016). These studies have shown encouraging results at the group level but also found large variability on the individual level. Here, we will assess the reliability of the suggested 4-choice fNIRS-BCI in six individual participants over the course of three fNIRS sessions across three consecutive days. Next to reliability over time, rehabilitation professionals have emphasized a need for BCIs to work reliably in different environments (Nijboer, 2015). The limited amount of studies that have tested an fNIRS-BCI in a non-laboratory environment, have usually done so in an environment familiar to the subject, for example their home or care center (Abdalmalak et al., 2017b; Borgheai et al., 2020; Li et al., 2021). However, a reliable fNIRS-BCI should also be able to perform in more noisy (social) environments. Therefore, two participants in our study will use the fNIRS-BCI in a cafeteria.

The simplicity of the temporal encoding paradigm developed in our lab (Nagels-Coune et al., 2017, 2020; Benitez-Andonegui et al., 2020) is enabled by straightforward univariate analysis – using only the information of the participant-specific most-informative fNIRS channel-by-chromophore. Despite the initial success in communication with ALS patients using a single-channel single-wavelength approach by Naito et al. (2007), BCI studies rarely decode information from a single channel (Benitez-Andonegui et al., 2020; Nagels-Coune et al., 2020). The majority of fNIRS-BCI studies use a large number of fNIRS channels and analyze data using a multivariate approach (Batula et al., 2014; Naseer and Hong, 2015a; Weyand and Chau, 2015; Hong et al., 2018). Large optode setups are generally experienced as uncomfortable and reports exist of participants withdrawing from fNIRS studies because of it (Suzuki et al., 2010; Cui et al., 2011; Rezazadeh Sereshkeh et al., 2018). Being able to use sparse channel setups would greatly increase clinical application and patient comfort. In addition, which chromophore, i.e., HbO or HbR, is most suited for BCI purposes is still a matter of debate. HbO is most often used in BCI because of its high amplitude (Leff et al., 2011), but HbR is thought to be less contaminated by physiological noise (Kirilina et al., 2012). Previous studies from our lab have reported a roughly comparable amount of participants in which HbO outperforms HbR and vice versa (Benitez-Andonegui et al., 2020; Nagels-Coune et al., 2020). In light of this, we focus our analyses in the current study on a participant-specific most-informative fNIRS channel-by-chromophore and compare it with results obtained from averages of two and three channels, to gauge the trade-off between number of optodes and decoding accuracy.

The aims of the current fNIRS-BCI study are: (1) to replicate the success of the temporal encoding paradigm using a four-choice paradigm, (2) to explore three different sensory encoding modalities, i.e., auditory, visual, and tactile, within the same participants employing an identical BCI paradigm, (3) to assess the reliability of the fNIRS-BCI across time and environments, and (4) to gauge the trade-off between number of optodes and decoding accuracy. To reach these aims, six participants answered four-choice questions using motor imagery, i.e., mental drawing. Motor imagery was guided by the auditory, visual or tactile sensory encoding modality. Each participant performed three fNIRS sessions on three consecutive days. Two participants were tested in an ecologically valid environment, i.e., a cafeteria, whereas the others were tested in a laboratory environment. Answer decoding was performed using a participant-specific most-informative fNIRS channel-by-chromophore. The possible advantage in terms of decoding accuracy of averaging two and three most-informative channels is explored. Finally, to capture the BCI users’ subjective experiences, we administered several in-house questionnaires that assess motor imagery skills, mental imagery strategies, easiness and pleasantness of the three sensory encoding modalities, and level of comfort during our study.

## Materials and Methods

### Participants

Eight participants were tested, of which two were excluded from this paper. One participant dropped out after session 2 due to personal matters, while a second participant was excluded following experimental error during the first recording session. The remaining six participants reported having no major disturbance of their visual, auditory or haptic capacities. The average age was 29.5 years (SD = 9.6) and all participants were right-handed females. Participants’ characteristics that were of interest for the current BCI study are listed in Table 1. Written informed consent was acquired from each participant at the beginning of the first fNIRS session. The experimental procedure conformed to the Declaration of Helsinki and was approved by the institutional review board. All participants were compensated with gift vouchers for their participation.

**TABLE 1:** Participant characteristics.

Participant	Age range	fNIRS-cap size	Previous BCI experience	Location	Motor imagery ability	fNIRS data analysis
P1	20–25	58	First time	Lab	13	*Post hoc*
P2	20–25	56	3–4 times	Lab	14	*Post hoc*
P3	25–30	58	First time	Lab	11	*Post hoc*
P4	20–25	58	First time	Lab	19	*Post hoc*
P5	45–50	56	>10 times	Cafeteria	19	Real-time
P6	25–30	56	>10 times	Cafeteria	19	Real-time

*The table shows for each participant the age range (years), fNIRS capsize (cm), BCI experience, location of the fNIRS sessions, self-reported motor imagery ability (0–20), and time point of fNIRS data analysis.*

### Location: Lab or Cafeteria

Four participants were measured in a laboratory setting (see Table 1). These participants were measured in a separate room that was completely dark during the fNIRS session. The experimenters could communicate with the participants via a speaker system. Two participants were measured in the university cafeteria. In this location, there was considerable background noise from students passing by or sitting at a nearby table. In both locations, an overcap/shading cap was placed over the fNIRS cap to shield the detectors from overexposure to outside light that could have otherwise saturated the optodes (Pinti et al., 2018a).

### Participant Preparation on Day 1

#### Motor Imagery Ability Questionnaire

Subjective reports of mental imagery ability have been found to correlate with objective measures of brain activation (Cui et al., 2007; Lorey et al., 2011; Ahn et al., 2018). In the current study, participants were asked to draw a rough sketch of a house, after which they were asked to imagine drawing the same sketch. They were encouraged to focus on the wrist and whole hand movements during the imagery period. Afterward they were asked to rate the following five features on a 5-point Likert scale: (1) vividness of their imaginary sketch, (2) similarity of their imaginary sketch to their real sketch, (3) ease of imagination while mental drawing, (4) their imaginary skills in general, and (5) enjoyment of the mental drawing task. This in-house questionnaire is based on existing questionnaires measuring related constructs (Barber and Wilson, 1978; Hall and Pongrac, 1983) and can be consulted in the Supplementary Material.

#### Autobiographical Questions

Autobiographical questions were created to ensure stability of answers over the three consecutive days. The six autobiographical questions can be consulted in the Supplementary Material. An example question is “Which country were you born in?” with the answer options being “Netherlands,” “Germany,” “Belgium,” or “Other.” The page with the “true answers” of the participant was kept in a closed envelope until the fNIRS data was analyzed. Experimenters were thus blind to the reported answers during the fNIRS sessions and *post hoc* analyses.

#### Participant Training

Participants were instructed to imagine drawing with their right hand with a comfortable and consistent speed for a duration of 10 s. We verbally suggested drawing simple contour images (e.g., a house, boat, and car) or small geometrical shapes (e.g., cubes, triangles, and circles). Participants chose their preferred image/shape, as the specific motor imagery content was not decoded by our BCI. If a mental image or shape was completed under 10 s, participants were instructed to recommence the mental drawing until they were cued to stop. During the rest periods (20 s), participants were instructed not to think about anything in particular and refrain from motor imagery. Participants were asked to practice mental drawing during three practice questions, with one question presented in each of the three sensory encoding modalities. The practice questions were selected from the autobiographical questions and followed an identical procedure to the answer-encoding runs elaborated on below (see Figure 1). The instructional part took around 15 min and the practice questions took around 5–10 min, depending on the participant. If the participant felt comfortable and had no more questions, the fNIRS cap was placed on the participant’s head and the first fNIRS run was conducted.

### fNIRS-Based Localization Procedure and Communication on Day 1, Day 2, and Day 3

**FIGURE 2:**
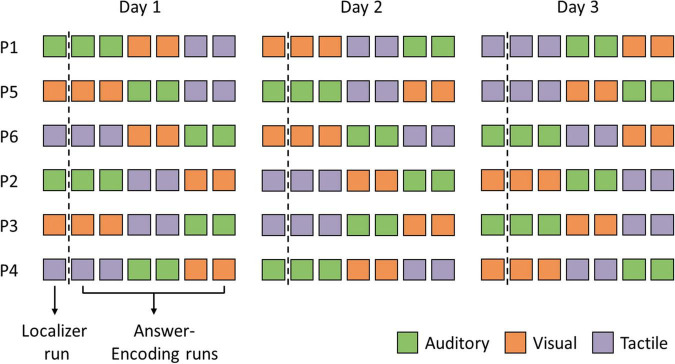
Participant-specific experimental protocols. Each fNIRS session consisted of seven functional runs: one localizer run and six answer-encoding runs. Note that the three sensory encoding modalities, i.e., auditory (green), visual (orange), and tactile (purple), were counterbalanced across participants and sessions.

Each fNIRS session consisted of seven functional runs: one localizer run and six answer-encoding runs. All fNIRS sessions were identical across the three testing days, with the questions and answer options presented in identical order, except for the sensory encoding modalities, which were counterbalanced across participants and sessions (see Figure 2). Software used for stimulus presentation were PsychoPy v.1.9 (Peirce, 2009) and NIRStim (v.3.0; NIRx Medical Technologies). Audio files were created using the text-to-speech function of NaturalReader^1^.

#### Localizer Run

The localizer run served to select a set of fNIRS channels for data analysis of the subsequently obtained answer-encoding data. After an initial 60 s rest period, the participant got the instruction to start mental drawing. After 10 s the participant got the instruction to stop mental drawing. After a rest period of 20 s, the participant was instructed to start the mental drawing again. Overall, 10 mental drawing trials with a duration of 10 s each were recorded. The total length of the localizer run was 6 min (60 s rest period + 10 × 10 s mental drawing + 10 × 20 s rest period). In the visual and auditory localizer, participants saw or heard the word “draw” and “rest.” In the tactile localizer, the experimenter touched the participant’s hand to signal start and stop of the mental drawing. A stroke across the participant’s hand signaled “draw,” whereas a soft tap on the hand signaled “rest.”

#### Answer-Encoding Runs

The six four-choice questions were posed in six answer-encoding runs in every fNIRS session. At the beginning of each answer-encoding run, there was a 60 s rest period. Then, the participant heard or saw the question (7 s), after which the four answer options (10 s each) were presented serially. The participant started mental drawing when she saw/heard/felt the cue for the answer option of their choice. Presentation of the following answer option (in the case of answer A, B, or C) or the cue for “rest” (in the case of answer D), signaled to the participant to stop performing mental imagery (see Figure 1). The four answer options were serially presented five times, resulting in five trials per question/answer-encoding run. An answer-encoding run took 6 min 7 s (60 s rest period + 7 s question + 5 × 40 s mental drawing + 5 × 20 s rest period). In the visual and auditory answer-encoding runs, participants read or heard the question, answer options and rest cue. In the tactile answer-encoding runs, the question and answer options were presented auditorily prior to the start of the run. The participant had to memorize the order of the answer options, as during the run no auditory instructions were given. The answer options were indicated by touching participant’s fingers. Pinkie finger indicated answer option A, ring finger corresponded to option B, middle finger to C and index finger to D. The beginning of the resting period was communicated through a stroke over the full hand. The two participants in the university cafeteria received feedback during the fNIRS sessions, where the experimenter communicated the decoded answers to the participant after each answer-encoding run.

#### Questionnaire of Strategy and Comfort

After each fNIRS session, participants filled in a short questionnaire in which they were asked to shortly describe and/or draw what they imagined. They were also asked to describe how they experienced the fNIRS-BCI session. Lastly, participants were asked to rate their general level of comfort, cap comfortability and tiredness on a 10-point Likert scale (1 indicating “uncomfortable/very tired” and 10 indicating “very comfortable/not tired at all”). The questionnaire of strategy and comfort is provided in the Supplementary Material.

### Questionnaire of General Study Impression on Day 3

On day 3, thus the last fNIRS session, participants filled in a final in-house questionnaire, which can be consulted in the Supplementary Material. This last questionnaire focused on participants’ motivation, general impression, their prior BCI experience, their mental imagery experience throughout the study and their emotions while using the BCI. Participants’ rated the easiness and pleasantness of the three sensory encoding modalities on a 10-point Likert scale (1 indicating “not pleasant/easy at all” and 10 indicating “very pleasant/easy”). Lastly, participants’ rated the three encoding modalities according to their relative liking (1 = best, 2 = medium, and 3 = worst).

### fNIRS Data Acquisition

**FIGURE 3:**
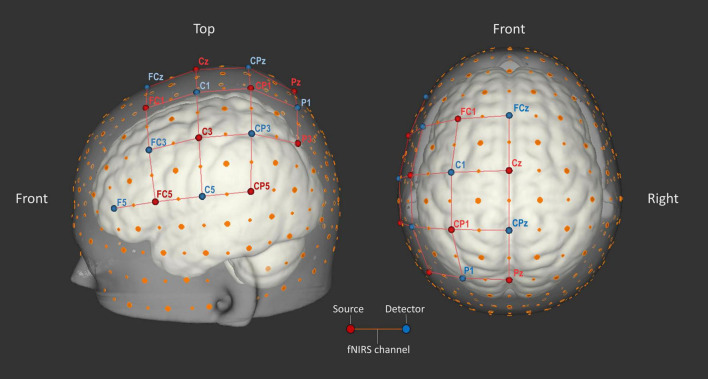
Optode layout. Eight source (red) and eight detector (blue) optodes were placed on 16 points according to the international 10–20 EEG system. Large orange dots represent reference points of the 10–20 system, whereas small orange dots represent reference points of the extended 10–10 EEG system (Oostenveld and Praamstra, 2001). The red lines represent 23 source-detector pairs (each forming an fNIRS channel). Image created using NIRSite software (v.1; NIRx Medical Technologies; RRID:SCR_002491).

Data was obtained with the continuous-wave NIRScout-816 system (NIRx Medical Technologies; RRID:SCR_002491) and was recorded using NIRStar software (v14.2 & v15.2; NIRx Medical Technologies; RRID:SCR_014540). Eight light source and eight light detector optodes were installed. Sources emitted light at wavelengths 760 and 850 nm, while detectors recorded the near-infrared light, which was sampled at a frequency of 7.8125 Hz. The optodes were placed in spring loaded optode holders attached to the cap. They were positioned on known markers from the international 10–20 EEG system (see Figure 3). The resulting 23 source-detector pairs, referred to as fNIRS channels, covered large parts of left-hemispheric fronto-parietal cortex. Frontal areas such as motor cortex (M1), supplementary motor area and premotor cortex are known for their activation during motor imagery (Porro et al., 1996; Sitaram et al., 2007; Pfurtscheller et al., 2008; Kanoh et al., 2009; Holper and Wolf, 2011; Abdalmalak et al., 2017a). Parietal areas, such as primary somatosensory cortex (S1) and intraparietal cortex, are also known to be activated by motor imagery (Fleming et al., 2010; Lorey et al., 2011; Aflalo et al., 2015). In two participants, P1 and P2, optodes forming channels that were not selected for subsequent analyses were physically removed after the localizer run to reduce possible participant cap discomfort.

### Data Analysis

#### fNIRS Signal Analyses

The main outcome of the current temporal encoding paradigm is communication accuracy, which was assessed as the percentage of correctly decoded answers. A set of signals-of-interest (SOIs), i.e., channel-by-chromophore combinations, were selected for each individual based on the participant’s localizer run’s data of that day. Answers were decoded from time courses of these SOIs with a univariate analysis using a General Linear Model (GLM) approach (Tak and Ye, 2014). The data of the four participants in the lab were analyzed *post hoc* in simulated real-time, whereas the data of the two participants in the cafeteria were analyzed online (i.e., intra-session).

##### Raw Signal Processing

Firstly, signal quality was checked for each channel in each fNIRS session. A channel-wise coefficient of variance percentage [CV%; see Piper et al. (2014) for a more detailed description] was calculated using the localizer run data and an in-house Matlab script. Channels with a CV% above 15 were deemed to have poor signal-to-noise ratio and were excluded from further analysis (Schmitz et al., 2005; Schneider et al., 2011; Piper et al., 2014; Pfeifer et al., 2018).

The raw signal from the remaining channels was processed using Turbo-Satori software (v1.6.4, Brain Innovation B.V., Maastricht, Netherlands). Baseline calculations were performed on the data of the first minute of each run. Linear trend removal and moving average filtering (low-pass cut-off frequency: 0.25 Hz, filter order: 2; high-pass cut-off frequency: 0.01 Hz, filter order: 1) were applied. GLM analyses were performed on the preprocessed signal. A linear confound predictor and a high-pass confound predictor (sine + cosine) with a cutoff frequency of 0.0002 Hz were included in the GLM to account for any residual slow drifts. Residuals were corrected for serial correlations (Luhrs and Goebel, 2017).

##### Signal-of-Interest Selection

A participant-specific most-informative channel-by-chromophore combination was chosen based on the localizer data of that day. Two GLMs were fitted, one applied to HbO data and the other to HbR data, using a model including only a single predictor for mental drawing. The predictor was convolved with a standard hemodynamic response function (HRF). The default HRF from SPM12 was used (two gamma HRF, the onset of response and undershoot 6 and 16 s, respectively, dispersion 1 s, response to undershot ratio 6). The same amplitudes were used for the HbO and HbR task predictors. The contrast “mental drawing vs. rest” was computed for each channel and chromophore. The channel-by-chromophore combination revealing the highest *t*-value of this contrast was chosen as the SOI (Benitez-Andonegui et al., 2020). In other words, different chromophores could be selected for different participants. This subject-specific channel-by-chromophore was considered for the answer decoding in the context of the first three aims of this study. For the fourth aim (effect of signal averaging), the 2nd best SOI and 3rd best SOI were identified through selection of the 2nd and 3rd highest *t*-value for the chosen chromophore.

##### Answer Decoding

The first trial of each run was discarded from the analyses as it served as a practice (“warm-up”) trial for the participant (Moriguchi and Shinohara, 2019; Techayusukcharoen et al., 2019; Li et al., 2021), resulting in four trials per answer-encoding run. Participants’ answers were decoded from the time course of the SOI by judging either each trial individually (single-trial analysis) or joint analysis of the four trials per answer-encoding run (multi-trial analysis). Four GLMs were fitted per trial (single-trial analysis) or per run (multi-trial analysis) using four reference-time courses. The reference-time courses correspond to the four answer-encoding options in our design (see Figure 1). The default HRF from SPM12 was used (for details see above). The same amplitudes were used for the HbO and HbR task predictors. The contrast “mental drawing vs. rest” was computed for the SOI. This resulted in four *t*-values based on the four time course predictors for each of the four answer options. The answer option for which the highest *t*-value was obtained was chosen as the decoded answer. In the context of our fourth research aim, i.e., levels of signal averaging, four GLMs were also fitted to the 2nd and 3rd most-informative signal. The contrast “mental drawing vs. rest” was computed for each SOI. The four resulting *t*-values of SOI1 and SOI2 were averaged (SOI1-2), as well as the four *t*-values of SOI1, SOI2, and SOI3 (SOI1-2-3). The answer option for which the highest *t*-value was obtained was chosen as the decoded answer.

For each participant 72 single-trial answers (4 trials × 6 answer-encoding runs × 3 sessions) and 18 multi-trial answers (6 answer-encoding runs × 3 sessions) were decoded. These decoded answers were then compared to the “true answers,” i.e., the answers participants noted down before the first session. Our main outcome measure, decoding accuracy (in%), was calculated for each participant by dividing the number of correct answers by the total amount of answers, i.e., 72 for the single-trial and 18 for the multi-trial approach. In the context of the research aims 2 and 3, i.e., exploring sensory encoding modality and reliability over time, the decoded answers were split in three groups, i.e., per modality (auditory, visual, and tactile) and per fNIRS session (day 1, day 2, and day 3). The number of correctly decoded answers was divided by the total amount of answers, i.e., 24 for the single-trial and 6 for the multi-trial approach, to attain decoding accuracies. Lastly, the group mean was calculated together with the standard deviation. In the context of research aim 4, i.e., different levels of signal averaging, all 72 single- and 18 multi-trial decoded answers were considered. Decoding accuracies, were calculated for each participant and for each level of signal averaging (SOI1, SOI1-2, and SOI1-2-3) by dividing the number of correct answers by the total amount of answers. Also here the group mean was calculated together with the standard deviation.

##### Chance Level Definition

The theoretical chance level of our four-choice BCI is 25%. However, given the limited amount of trials within a single participant, common in BCI studies, a threshold based on binomial distribution is considered more trustworthy and therefore more frequently used (Noirhomme et al., 2014). To assess the significance of each participants’ decoding accuracy in the current study, chance levels were calculated based on a binomial distribution. The number of independent outcomes was four (*k* = 4) and the significance level was set at 5% (α = 0.05). For the single-trial results the number of independent trials was 72 (*n* = 72), resulting in the upper-bound chance level of 33.33%. In other words, if 24 or more trials out of 72 were decoded correctly this was considered a significant result. For the multi-trial results the number of independent trials was 18 (*n* = 18), resulting in an upper-bound chance level of 44.44%. If 8 or more trials out of 18 were decoded correctly this was considered a significant result. The chance levels of 33.33% (single-trial) and 44.44% (multi-trial) were used to evaluate the general decoding accuracies (aim 1) and the effect of signal averaging (aim 4). For evaluation of the participants’ decoding accuracies per sensory encoding modality (aim 2) and per fNIRS session (aim 3), the chance level was 41.67% (single-trial, *n* = 24), and 50% (multi-trial, *n* = 6).

### Subjective Measures

The ratings on the five features of the motor imagery ability questionnaire were summed to obtain a single motor imagery ability score, with a maximum score of 20. Two Pearson correlation coefficients (α = 0.05) were computed to assess the relationship between the motor imagery ability score and single- and multi-trial decoding accuracies, both decoded from the single most-informative channel-by-chromophore. All remaining in-house questionnaires are reported on a descriptive level, i.e., sample average and standard deviation ($\bar{X}$ ± SD), given our small sample size.

## Results

### Signal of Interest Selection

**FIGURE 4:**
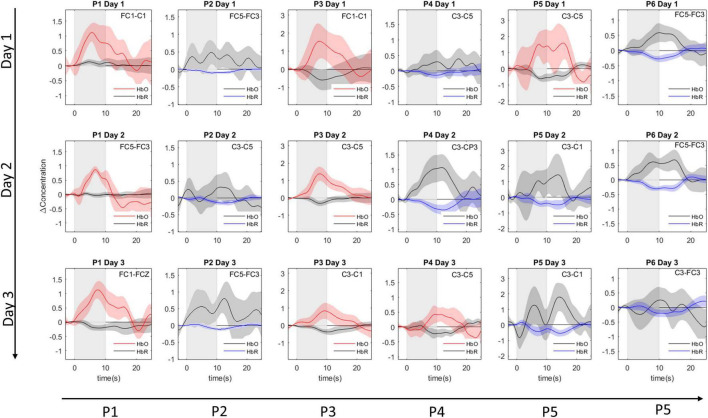
Subject specific event-related averages. Event-related averages for the most-informative channel-by-chromophore combination for each participant (columns) in each session (rows). The shaded rectangle represents the mental task duration (10 s). The event-related averages are depicted from 3 s before until 15 s after mental task performance. The selected channel can be read in the right upper corner. Colored lines depict the average concentration change of the selected chromophore: red for oxygenated hemoglobin (HbO) and blue for deoxygenated hemoglobin (HbR). For completeness, gray lines depict the chromophore counterpart belonging to the most-informative channel. The shaded area around the mean average line represents the 95% confidence interval of the mean.

All channels had sufficient signal quality, with a CV% below 15 across the three sessions. For each participant a single best-suited channel-by-chromophore combination was selected (see Supplementary Figure 1 and Supplementary Table 1). In two participants, an HbO channel was the most informative channel across all sessions. In another two participants, HbR was the most informative chromophore across all sessions. In the remaining two participants, either HbO or HbR was selected depending on the session. The event-related averages of the chosen channel-by-chromophore combinations are shown in Figure 4. The most informative fNIRS channels across all participants and sessions were FC5-FC3 and C3-C5, both chosen in five out of 18 cases (six participants × three fNIRS sessions). In the Supplementary Figure 2 and Supplementary Table 2, detailed information on the channel selection frequency for the 1st, 2nd, and 3rd most informative channel selection is provided.

### Mean Answer-Decoding Accuracies

**FIGURE 5:**
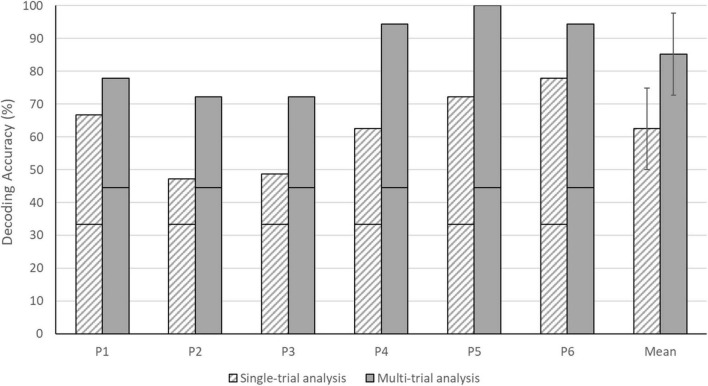
Single- and multi-trial answer decoding accuracies for individual participants and the group. The black horizontal stripe within each bar graph represents the chance level for single-trial (33.33%) and multi-trial (44.44%) accuracies. The error bars depict the standard deviation of the group mean. Note that all participants performed above the chance level in both analyses.

Each of the six individual participants reached a decoding accuracy significantly above the chance level for both single- and multi-trial analyses (see Figure 5). As expected, the multi-trial answer decoding outperformed the single-trial approach in each participant. Individual single-trial decoding accuracies ranged from 47.22 to 77.78%, whereas multi-trial decoding accuracies ranged from 72.22 to 100.00%. The group mean of the single-trial approach was 62.50% (SD = 12.42), whereas the multi-trial group mean was 85.19% (SD = 12.51). Note that all answers of participant 5 were decoded correctly across the three sessions using the multi-trial analysis, resulting in a 100% accuracy. For participants 4 and 6, 17 out of 18 answers were decoded correctly using the multi-trial analysis, resulting in a 94.44% accuracy.

### Answer-Decoding Accuracies Across Sensory Encoding Modalities

**FIGURE 6:**
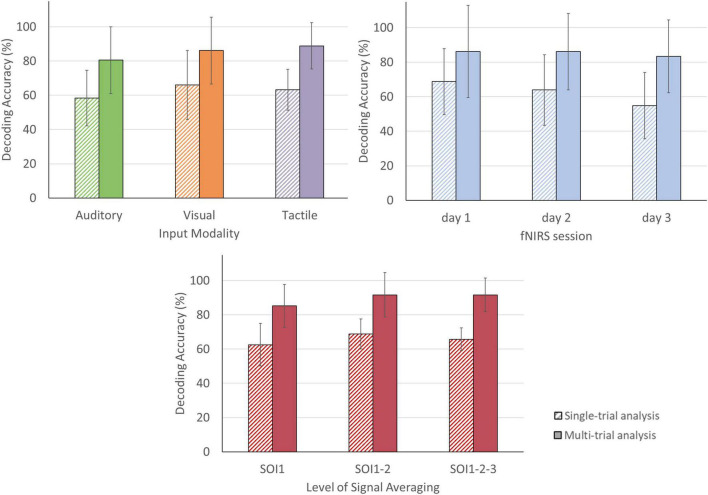
Single- and multi-trial group mean answer decoding accuracy per sensory encoding modality, fNIRS session and level of signal averaging. Accuracies are depicted for the single-trial (striped bars) and multi-trial (solid bars) analysis. **Top left**: Accuracies according to sensory encoding modality. Note that all sensory encoding modalities were effective. **Top right**: Accuracies according to fNIRS session. Note that the multi-trial accuracies remained relatively stable over the three fNIRS sessions. **Bottom**: Accuracies according to level of signal averaging. Note that averaging across two (SOI1-2) or three (SOI1-2-3) signals slightly outperforms the single channel-by-chromophore approach (SOI1). The error bars depict the standard deviation of the group mean. Abbreviations: SOI, signal-of-interest.

**FIGURE 7:**
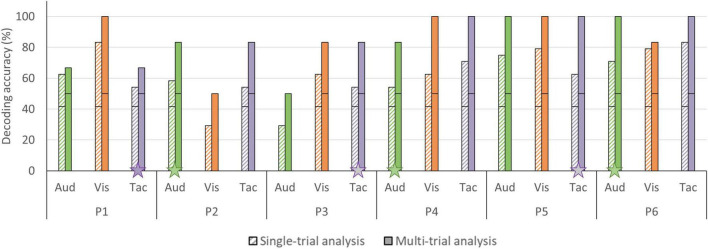
Single- and multi-trial individual decoding accuracies per sensory encoding modality. Individual decoding accuracies for the auditory (green), visual (orange), and tactile (purple) encoding modality obtained with single-trial (striped bars) and multi-trial (solid bars) analysis. The black horizontal stripe within each bar graph represents the chance level for single-trial (41.67%) and multi-trial (50.00%) accuracies. The stars on the horizontal axis mark participants subjectively preferred sensory encoding modality. Abbreviations*:* Aud, auditory; Vis, visual; and Tac, tactile.

In the single-trial approach, mean accuracies of 58.33% (SD = 16.24) were obtained for the auditory, 65.97% (SD = 20.14) for the visual and 63.19% (SD = 11.91) for the tactile modality (see Figure 6). In four participants, decoding accuracies obtained with each of the three sensory encoding modalities were significant (chance level of 41.67%). In two participants, one encoding modality did not reach significance (visual [P2], auditory [P3]; see Figure 7). In the multi-trial approach, mean accuracies increased to 80.56% (SD = 19.48) for the auditory, 86.11% (SD = 19.48) for the visual and 88.89% (SD = 13.61) for the tactile modality (see Figure 6). In all participants, accuracies with respect to the three encoding modalities reached or surpassed the chance level of 50% (see Figure 7). In one participant, participant 5, all three encoding modalities reached 100% accuracy.

### Answer-Decoding Accuracies Across Time

**FIGURE 8:**
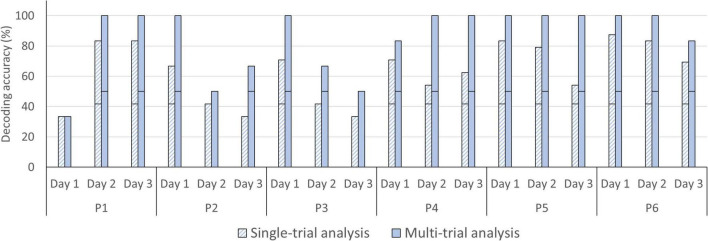
Single- and multi-trial individual decoding accuracies across fNIRS session. Individual decoding accuracies for the 1st fNIRS session (day 1), 2nd fNIRS session (day 2), and 3rd fNIRS session (day 3) obtained with single-trial (striped bars) and multi-trial (solid bars) analysis. The black horizontal stripe within each bar graph represents the chance level for single-trial (41.67%) and multi-trial (50.00%) accuracies.

For the single-trial decoding accuracies, a slightly declining trend can be observed, with 68.75% on day 1 (SD = 19.14), 63.89% on day 2 (SD = 20.36), and 54.86% on day 3 (SD = 19.26; see Figure 6). In three participants, accuracies were significant in all three fNIRS sessions (chance level of 41.67%). In the three remaining participants, one fNIRS session did not reach significance (session 1 [P1], session 3 [P2 and P3]; see Figure 8). In the multi-trial approach, group mean decoding accuracies remained relatively stable across the three consecutive fNIRS sessions, with 86.11% on day 1 (SD = 26.70), 86.11% on day 2 (SD = 22.15) and 83.33% on day 3 (SD = 21.08; see Figure 6). In all five participants, the three fNIRS sessions reached or surpassed the empirical chance level of 50% (see Figure 8). In one participant, one fNIRS session did not reach significance (session 1 [P1]).

### Answer-Decoding Accuracies Across Different Degrees of Channel Averaging

**FIGURE 9:**
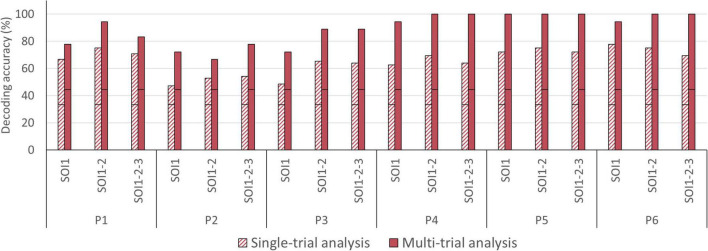
Single- and multi-trial individual decoding accuracies across level of signal averaging. Individual decoding accuracies for the most informative channel-by-chromophore (SOI1), the average of the two most informative channel-by-chromophore (SOI1-2) and the average of the three most informative channel-by-chromophore (SOI1-2-3) obtained with single-trial (striped bars) and multi-trial (solid bars) analysis. The black horizontal stripe within each bar graph represents the chance level for single-trial (33.33%) and multi-trial (44.44%) accuracies.

In the single-trial approach, decoding accuracies improved slightly when averaging two or three channels, from 62.50% [SOI1; SD = 12.42] to 68.75% [SOI1-2; SD = 8.77] and 65.74% [SOI1-2-3; SD = 6.67], compared to when analyzing a single channel-by-chromophore (see Figure 6). In five participants, namely P1, P2, P3, P4, and P5, averaging across two or three channels resulted in an improved decoding accuracy (see Figure 9). In the multi-trial approach, decoding accuracies also increased slightly when averaging across two or three channels from 85.19% [SOI1; SD = 12.51] to 91.67% [SOI1-2; SD = 13.03] and 91.67% [SOI1-2-3; SD = 9.78]; see Figure 6). In four participants, namely P1, P3, P4, and P6 channel averaging with either two or three channels improved decoding accuracy (see Figure 9). In one participant, P5, the single channel multi-trial decoding accuracy was already perfect and hence channel averaging could not further improve this score.

### BCI User Experience

#### General BCI Experience

Four participants chose to mentally draw a house. One participant chose to mentally draw a house with a tree next to it and another participant imagined drawing small cubes. Participants felt generally comfortable during the fNIRS sessions (rating 7.72/10 ± 1.53). All participants reported feeling confident using the system. No participant reported feeling anxious. The fNIRS cap with spring-loaded optodes was experienced as reasonably comfortable (rating 6.72/10 ± 2.32), with 5 out of 6 participants reporting to have felt comfortable using the system. Two participants did report some discomfort during a single fNIRS session, with a few of the optodes causing noticeable pressure on the head. Participants did not experience significant fatigue (6.28/10 ± 1.93) during the experiment. Moreover, general comfort, cap comfort and fatigue remained relatively stable over the three fNIRS sessions (see Supplementary Figure 3). None of the participants reported a lowering motivation over the course of the fNIRS sessions.

#### General and Individual Preference of Sensory Encoding Modality

**FIGURE 10:**
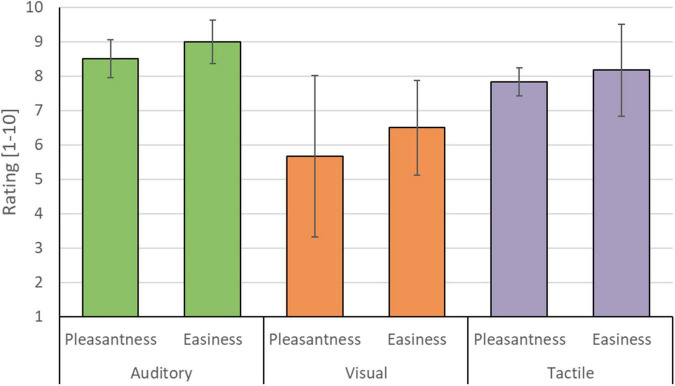
Mean participant rating of perceived pleasantness and easiness for each of the three sensory encoding modalities. Participants rated the auditory (green), visual (orange), and tactile modality (purple) on a scale from 1 (not pleasant/easy at all) to 10 (very pleasant/easy). Error bars depict the standard deviation of the group mean. Note that the auditory and tactile modality were rated as relatively more pleasant and easy compared to the visual modality.

The auditory modality was judged as being the most pleasant (8.50/10 ± 0.55) and easy (9.00/10 ± 0.63) sensory encoding modality, followed by the tactile modality (pleasantness 7.83/10 ± 0.41; easiness 8.16/10 ± 1.33). Participants’ judgment with respect to the visual modality were generally lower (pleasantness 5.60/10 ± 2.34; easiness 6.50/10 ± 1.38) and less in agreement, as reflected in a relatively large standard deviation (see Figure 10). Three participants preferred the auditory encoding modality, whereas the other three participants preferred the tactile encoding modality. No participant preferred the visual encoding guidance. Four participants indicated in the remarks section that not being able to close their eyes hindered performing mental imagery. Participant 5 and 6 were measured in a naturalistic environment and both indicated that the auditory and tactile runs were more pleasant/relaxing than the visual runs because they could either look around or close their eyes, instead of having to fixate on the screen. P2 and P3 had identical multi-trial accuracies in the tactile modality (see Figure 7) but expressed differential subjective experiences. While P2 indicated that she became more uncomfortable during the tactile runs due to the presence of an experimenter, P3 felt more confident and reassured with the experimenter present.

#### Motor Imagery Ability Questionnaire

Participants’ mental drawing was generally vivid (rating 3.33/4 ± 0.82) and judged similar to their sketch (3.17/4 ± 0.98). They found the mental drawing task easy (3.33/4 ± 0.82) and enjoyable (2.83/4 ± 0.75). Participants judged their general imagination as being good (3.17/4 ± 0.98). The total scores on the motor imagery ability questionnaire (15.83/20 ± 3.60) are reported in Table 1. Self-reported motor imagery ability correlated significantly with the multi-trial decoding accuracies [*r*(4) = 0.95; *p* < 0.01], but not with the single-trial decoding accuracies [*r*(4) = 0.73; *p* = 0.10; see Supplementary Figure 4].

## Discussion

The results show that the temporal answer-encoding paradigm, recently developed by our group (Nagels-Coune et al., 2017, 2020; Benitez-Andonegui et al., 2020), is a an effective and convenient paradigm. Using a simple motor imagery task, relatively little preparation time and only a single fNIRS channel-by-chromophore combination, the paradigm enables effective and efficient four-choice BCI-based communication. Moreover, it is highly flexible as it allows for exploiting three different sensory encoding modalities (auditory, visual and tactile) for guiding answer encoding. Visual and auditory answer encoding in fNIRS-BCIs have been reported previously (Naito et al., 2007; Naseer and Hong, 2015a; Nagels-Coune et al., 2017, 2020; Benitez-Andonegui et al., 2020) but tactile guidance was explored here for the first time, with encouraging results. Moreover, the results based on six participants demonstrate reliable communication over the course of three consecutive days. Note that two of the six participants were tested under more ecologically valid conditions in a university cafeteria (vs. in a laboratory). In the following sections, the implications of the current study will be discussed in more detail, followed by limitations of the current and recommendations for future work.

### Temporal Answer Encoding – An Effective and Convenient BCI Paradigm

Many fNIRS-BCI studies have exploited the differential spatial brain activation patterns associated with the execution of two or more mental imagery tasks (Batula et al., 2014; Naseer and Hong, 2015a,b; Li et al., 2021). In previous work we combined the spatial features with a temporal component in the context of a binary fNIRS-BCI (Nagels-Coune et al., 2020). The decoding accuracy reached 66.67% (HbO) and 58.33% (HbR), with a subset of participants merely relying on the temporal aspect. Benitez-Andonegui et al. (2020) followed up with a six-choice fNIRS-BCI based on a purely temporal encoding (i.e., using only one mental imagery task), reaching an accuracy of 73.96%. In the current experiment, the temporal answer-encoding paradigm was tested in the context a four-choice fNIRS-BCI (see Figure 1). The single-trial decoding accuracy of 62.50% (see Figure 5) obtained in this work is decent, given that other 4-choice fNIRS-BCI applications reached single-trial accuracies of 45.7% (Batula et al., 2014) and 73.3% (Naseer and Hong, 2015a). Note, however, that in both of these studies, large arrays of 18 (Batula et al., 2014) and 32 (Naseer and Hong, 2015a) fNIRS optodes were used to discern differentiable spatial brain activation patterns between four imagery tasks. In the current study, a single channel-by-chromophore was analyzed and participants performed a single imagery task. We found an average multi-trial decoding accuracy of 85.19%, with each individual participant showing significant decoding accuracies (see Figure 5). One participant, P5, even reached 100% decoding accuracy across the three fNIRS sessions. A joined analysis of several encoding trials per answer (multi-trial analysis) substantially increased the decoding accuracy, as has been reported previously (Nagels-Coune et al., 2017, 2020; Benitez-Andonegui et al., 2020).

The temporal answer-encoding paradigm presented here has many advantages due to its simplicity. Firstly, participants use a single mental imagery task, which reduces working memory load. Secondly, there is no need for a lengthy period for training a classifier, given that decoding analyses rely on straightforward GLM analysis (see section “Materials and Methods”). In the current experiment, 6 min localizer runs were performed by each participant to identify channels of interest. Note that in EEG-BCIs, users often need considerably longer training periods to be able to control their brain rhythms (Pires et al., 2012), whereas in multivariate fNIRS-BCIs classifiers need extensive training datasets (Batula et al., 2014; Naseer and Hong, 2015a). However, a possible disadvantage of the temporal encoding paradigm, compared to multivariate classification methods, is that answer-encoding trials tend to take more time. For example, in the four-choice fNIRS-BCI study by Naseer and Hong (2015a) a single trial lasted only 10 s (compared to 40 s in the current study). Thirdly, we show here that, in principle, the information obtained from a single fNIRS channel-by-chromophore combination suffices for successfully using the developed fNIRS-BCI. Note that in two participants many of the optodes were removed after the localizer run that served to identify the most-informative channel. The possibility to rely on a single fNIRS channel increases the comfortability of the fNIRS-BCI and the overall esthetics, factors often overlooked but being vital for the technology acceptance by users (Nijboer, 2015). Moreover, use of a small optode array could pave the way to cost reduction of fNIRS hardware (Tsow et al., 2021). Fourthly, encoded answer/commands can be decoded relatively easily in real-time with a basic GLM approach. In the current study, two participants received immediate feedback on their decoded answers. By using an existing commercially available software, here the Turbo-Satori software (Luhrs and Goebel, 2017) that particularly focuses on usability, we are one step closer to an fNIRS-BCI manageable by even caregivers and family members themselves. With this work, we further encourage fNIRS-BCI researchers to exploit the temporal features of the fNIRS signal for information encoding, next to using the spatial fNIRS-signal features that have been used so far (Batula et al., 2014; Naseer and Hong, 2015a; Weyand and Chau, 2015; Hong et al., 2018). The further exploration of a wide variety of paradigms might be necessary when taking into account the heterogeneous population of patients in need of a BCI.

### The Alternative Use of Different Sensory Encoding Modalities – A Promise for Clinical Applications

Most fNIRS-BCIs using mental imagery have used either the visual or auditory modality to guide answer encoding (Naito et al., 2007; Naseer and Hong, 2015a; Abdalmalak et al., 2017b; Nagels-Coune et al., 2017, 2020; Benitez-Andonegui et al., 2020). However, many of the target users have their vision affected (Gill-Thwaites and Munday, 2004; Riccio et al., 2012; Rousseau et al., 2012). Even target users with intact vision might prefer alternative encoding modalities, as a screen might exclude the user from ongoing social interactions (Nijboer et al., 2014). The current work reported the first multimodal fNIRS-BCI that alternatively incorporated auditory, visual and tactile sensory encoding within one experimental paradigm. Both the single- and multi-trial decoding accuracies were found to be above chance, being 58.33 and 80.56% for auditory, 65.97 and 86.11% for visual and, 63.19 and 88.98% for tactile encoding, respectively, (see Figure 6). These accuracies suggest that the fNIRS-BCI can work effectively with different sensory encoding modalities in healthy participants. Depending on the specific needs and preferences of potential patient BCI users, promising sensory encoding modalities can be selected. In the current work, three participants preferred the auditory modality, whereas the other three participants preferred the tactile modality (see Figure 7). Subjective preference can aid researchers to select a subject-specific optimal sensory encoding modality. For example, P2 had an identical multi-trial decoding accuracy using the auditory and tactile encoding modality, but subjectively preferred the auditory modality to guide motor imagery (see Figure 7). In the case of P2, choosing the auditory sensory encoding modality for future fNIRS-BCI use is an optimal decision.

To our knowledge, the current work is the first exploration of a tactile fNIRS-BCI. An experimenter stroked the BCI user’s fingers and hand at specified times to cue possible on- and offsets of mental imagery. Using this basic approach, each individual participant reached significance in the multi-trial analysis (see Figure 7). Tactile stimulation provided by a person can be considered an advantageous technical simplification, but one could also easily use an electric stimulation device to administer the tactile guidance (Lugo et al., 2014; Guger et al., 2017). Such experimental decisions might also depend on the subjective experience of the BCI user. In our study, participants expressed differential subjective experiences with the tactile encoding modality. While P2 indicated feeling more uncomfortable during the tactile runs due to the presence of an experimenter, P3 felt more confident and reassured with the experimenter in the room. A recent EEG-BCI study found that social presence and emotional support can enhance BCI accuracy for non-autonomous people, i.e., people that prefer to work in group (Pillette et al., 2020).

Participants generally experienced the auditory sensory encoding modality as pleasant (8.50/10 ± 0.55) and easy (9.00/10 ± 0.63), followed by the tactile modality (pleasantness 7.83/10 ± 0.41; easiness 8.16/10 ± 1.33) and lastly the visual modality (pleasantness 5.60/10 ± 2.34; easiness 6.50/10 ± 1.38). Participants reported being hindered by the constraints of the visual encoding in terms of concentration/fixation on the screen. This could be due to visual fatigue, as well as general annoyance of feeling “not socially present” (Nijboer et al., 2014). Another possible factor is the orthogonality of the sensory encoding modalities with respect to the mental task. The mental imagery task used here, i.e., mental drawing, is partly based on visual imagination. Therefore, auditory or tactile instruction modalities might have been experienced as less hindering of the – partly visual – motor imagination.

### A Reliable fNIRS-BCI Over Time and Environments

Most fNIRS-BCI studies were performed in a single session (Naito et al., 2007; Naseer and Hong, 2015a; Abdalmalak et al., 2017b; Nagels-Coune et al., 2017, 2020; Benitez-Andonegui et al., 2020) but effectiveness over time is a crucial factor for end users. The findings here show that our fNIRS-BCI works reliably over the course of three consecutive days, with multi-trial accuracies of 86.11% on day 1, 86.11% on day 2, and 83.33% on day 3 (see Figure 6). In the single-trial decoding accuracies a slightly declining trend can be observed, with 68.75% on day 1, 63.89% on day 2, and 54.86% on day 3 (see Figure 6). Although participants reported no decline in motivation across the fNIRS sessions, it is plausible that use of the fNIRS-BCI was less exciting on day 3. Therefore, participants might have been less focused on the task at hand in the final session. The only other longitudinal fNIRS-BCI study also reported no decline in BCI performance by an ALS patient (Borgheai et al., 2020).

Next to reliability over time, rehabilitation professionals have emphasized a need for BCIs to work reliably in different environments (Nijboer et al., 2014). Most fNIRS-BCIs that were tested outside the laboratory took place in a familiar and calm location such as the home or a care center (Abdalmalak et al., 2017b; Borgheai et al., 2020; Li et al., 2021). However, people with severe disabilities may leave their home and need to be able to communicate in varying contexts (Nijboer et al., 2014). Given the mobility of fNIRS hardware and its relative robustness against user head motion, fNIRS-BCIs may provide a useful opportunity in this context. In the current work, an fNIRS-BCI was tested in two healthy participants, P5 and P6, in a noisy and public place, which led to multi-trial accuracies of 100 and 94.44% (see Figure 5). These results are relatively high in the BCI field, where 70% accuracy is a common criterion in binary studies (Kubler et al., 2006). Note, however, that both participants had ample prior BCI experience (see Table 1) which might have facilitated their high accuracies. In addition, these participants received online feedback on the decoded answer, which might have had a beneficial effect on the participants’ general motivation. Participants that are more engaged in task performance are thought to produce more robust brain signals in a context of BCI (Nijboer et al., 2008, 2010). Given these encouraging results in two participants, future research may further explore the use of fNIRS-BCIs in more ecologically valid environments.

### Decoding From a Single Channel or Multiple Channels? – An Individual Matter

As a first approach, answer decoding was based on information obtained from a single fNIRS channel-by-chromophore combination. As in previous work from our group (Nagels-Coune et al., 2017, 2020; Benitez-Andonegui et al., 2020), we found that the most-informative chromophore is subject-specific. While selection of the most-informative chromophore, i.e., HbO or HbR, was quite stable within four subjects, for two participants the selected chromophore varied across sessions. The latter might be caused by the fact that fNIRS-cap placement (although performed as precise and consistent as possible across fNIRS sessions) might still result in inevitable variation of optode location. Another cause for variation in the selected chromophore might be the presence of physiological noise, which might differ across participants and even days. Currently there is no consensus that one chromophore outperforms the other in terms of signal quality (Kohl et al., 2020). Considering both chromophores, which is rarely done in fNIRS-BCI’s, seems the fair route until intensive investigation favors one chromophore over the other. This reasoning and our observations motivated to individually determine the best channel-by-chromophore combination per communication session.

We further investigated whether averaging two or three most informative fNIRS channels (compared to using a single channel-by-chromophore) improves answer-decoding accuracy. For participants showing high decoding accuracies using the single most-informative channel-by-chromophore, decoding improvement was marginal, possibly reflecting a ceiling effect. However, in participants with initially lower accuracies, averaging across channels revealed to have benefits (see Figure 9). For example, in P3 accuracy rose from 48.61% (SOI1) to 65.28% (SOI1-2) and 63.89% (SOI1-2-3). Averaging across a small number of channels in close proximity has been reported to result in more reliable measures (Wiggins et al., 2016). Future work should therefore investigate the accuracy benefit of adding a small number of channels in a systematic manner. We expect that channel averaging might be especially beneficial in cases where the single-channel fNIRS-BCI has low accuracy. A promising resource to ensure that the informative, here mental task sensitive, region is sampled by a small set of optodes is the Array Designer Toolbox (Brigadoi et al., 2018). Through automated optode array design for a specific region-of-interest, there is an increased cortical sensitivity compared to manual optode placement. Alternatively, if anatomical fMRI data is available, probabilistic maps of fMRI-activation from an independent dataset can guide optode placement (Benitez-Andonegui et al., 2021).

### BCI User Experience – A Factor Not to Be Overlooked

In the developing field of fNIRS-BCI, much of the published work has focused on methodological/technical development. Yet, the success of an fNIRS-BCI also relies heavily on the ability of the participant to produce robust and reliable hemodynamic signals. We administered several in-house questionnaires to explore user skills and experience, as these factor may influence the quality of the evoked fNIRS signals (Holper et al., 2011) and therewith BCI decoding accuracy (Cui et al., 2007; Weyand and Chau, 2015; Jeunet et al., 2017; Sargent et al., 2018). In addition, user experience affects the likelihood patients will actually use a BCI in a regular manner (Nijboer, 2015). Participants generally felt comfortable and motivated in our study. Two participants did experience some discomfort in one session due to pressure induced by a few optodes. Discomfort is not uncommon in fNIRS studies (Suzuki et al., 2010; Cui et al., 2011; Rezazadeh Sereshkeh et al., 2018) and constitutes another motivation to move toward small-scaled fNIRS-optode setups. General comfort, cap comfort and fatigue scores remained relatively stable over the three fNIRS sessions (see Supplementary Figure 3). Participants that rated their motor imagery ability as high, tended to have a high multi-trial decoding accuracy [*r*(4) = 0.95; *p* < 0.01; see Supplementary Figure 4]. In other words, participants that rated their imagination as vivid, similar to actual drawing, easy, enjoyable and generally good tended to achieve higher answer-decoding accuracies. This finding is in line with several BCI studies using EEG, fMRI and fNIRS neuroimaging techniques (Lorey et al., 2011; Jeunet et al., 2015; Weyand and Chau, 2015; Ahn et al., 2018). This link between mental task ability and BCI accuracy paves the way to mental imagery user training, especially in users with low BCI accuracy (Kaiser et al., 2014).

### Limitations and Future Work

A drawback of the current study is the absence of correction for physiological noise through the use of short-separation channels (Brigadoi and Cooper, 2015). Moreover, correction though spatial filtering was not possible since these approaches require coverage of a larger area than the region of interest (Zhang et al., 2016). Removal of systemic noise would likely have improved the reliability and accuracy of our BCI paradigm (Wiggins et al., 2016). However, through our focus on a participant-specific and daily-defined channel-by-chromophore, we did indirectly exclude “noisy” signals. For example, the event-related potential for P2 on day 1 (see Figure 4) shows a contaminated HbO signal and a clean HbR signal. Despite the relatively modest amplitude in HbR, compared to HbO, the HbR chromophore is chosen as the signal-of-interest.

Another drawback is the limited sample size in the current study. Generalization to the overall population is difficult based on the results of this sample. Nevertheless, the results are encouraging and show that four-choice fNIRS-based communication using different sensory encoding modalities is feasible. A more elaborate study with a larger sample size should be conducted following this proof-of-concept study.

Although our temporal encoding paradigm is effective, with 6 min 7 s per four-choice question, the information transfer rate is low. Three participants, P4, P5, and P6, had a significant single-trial decoding accuracy in each fNIRS session (see Figure 8). This finding suggest that robust communication is possible through joint analysis of less than four trials in some participants. Moreover, in these three subjects single-trial communication is already feasible with a decrease in decoding accuracy as the cost. Future fNIRS-BCI studies could improve the information transfer rate through shortening the mental task duration. In the temporal encoding six-choice fNIRS-BCI by Benitez-Andonegui et al. (2020) a mental task duration of 6 s yielded promising results. Another step toward drastically shortening encoding times could be to focus on the initial dip of the hemodynamic response, rather than the full response. Khan and Hong (2017) reached a 76.5% four-command decoding accuracy with their fNIRS-BCI with a post-stimulus window size of 2 s. Borgheai et al. (2020) also reported successful single-trial classification using a post-stimulus window size of under 4 s in ALS patients. Both studies highlight the efficacy of short event-related hemodynamic changes. Moreover, through such short post-stimulus windows, also the inter-stimulus interval can be shortened significantly. Future fNIRS-BCI development should further investigate and replicate these promising findings, as they would greatly enhance potential for daily use.

Furthermore, focusing on individualization of BCI procedures is highly recommended with respect to both the choice of the sensory encoding modality and the selection of a mental task to control the BCI. In the current study, all participants performed motor imagery, but other types of mental imagery should be explored as well, for example somatosensory imagery as recently applied in an fMRI-BCI context (Kaas et al., 2019). In an ideal case, a mental task should be individually chosen according to the BCI user’s preference from a compilation of proven BCI-control tasks (Weyand et al., 2015).

The fNIRS hardware used in the present study was rather bulky and transported on a cart, as is the case in most fNIRS studies (Scholkmann et al., 2014). However, recently developed mobile devices that can fit in a backpack (Pinti et al., 2018a), combined with a limited optode setup as proposed here, can result in a small-scaled fNIRS-BCI. These simplifications in hardware might further stimulate exploration of fNIRS-BCIs in ecologically valid environments. This would increase the chance that fNIRS-BCIs will be once indeed be used on a regular basis by patients, that are often already surrounded by bulky medical equipment (Nijboer et al., 2014).

## Conclusion

In the current study, we tested a four-choice multimodal fNIRS-BCI in six healthy subjects. Using a temporal encoding paradigm and decoding the answers from a single channel-by-chromophore time course resulted in mean single- and multi-trial decoding accuracies of 62.50 and 85.19%, respectively. Answer encoding was alternatively guided by three different sensory encoding modalities (visual, auditory, or tactile). Decoding accuracies were found to be stable across three consecutive days. Moreover, decoding accuracies from two experienced BCI users were stable in an ecologically valid setting, i.e., a cafeteria. Averaging of two or three most-informative channels further increased decoding accuracy compared to the single channel-by-chromophore approach. Future fNIRS-BCI studies should focus on increasing efficiency (e.g., by decoding from quick-to-detect features of the hemodynamic response, such as the initial dip) and on reporting relevant user experience.

## Data Availability Statement

The raw data supporting the conclusions of this article will be made available by the authors, without undue reservation.

## Ethics Statement

The study involving human participants was reviewed and approved by the institutional review board (Ethical Review Committee Psychology and Neuroscience, ERCPN – reference 180_07_06_2017) of the Faculty of Psychology and Neuroscience, Maastricht University. The participants provided their written informed consent to participate in this study.

## Author Contributions

BS, LN-C, and SK conceived and designed the study. LN-C, SK, BS, and AB-A obtained the data. LN-C performed the data analysis with the aid of BS, AB-A, and ML. PD, LR, and RG oversaw the analyses. LN-C wrote the first draft of the manuscript. All authors contributed to manuscript revision, read and approved the submitted version.

## Conflict of Interest

ML and RG are employed by the research company Brain Innovation B.V., Maastricht, Netherlands. The remaining authors declare that the research was conducted in the absence of any commercial or financial relationships that could be construed as a potential conflict of interest.

## Publisher’s Note

All claims expressed in this article are solely those of the authors and do not necessarily represent those of their affiliated organizations, or those of the publisher, the editors and the reviewers. Any product that may be evaluated in this article, or claim that may be made by its manufacturer, is not guaranteed or endorsed by the publisher.

## Footnotes

1
http://naturalreaders.com

## Abbreviations

SOI: signal-of-interest.
