# Supplementary material for: See, Hear, or Feel – to Speak: A Versatile Multiple-Choice Functional Near-Infrared Spectroscopy-Brain-Computer Interface Feasible With Visual, Auditory, or Tactile Instructions
Source: Front Hum Neurosci. 2021 Nov 25;15:784522. doi: 10.3389/fnhum.2021.784522 (PMC8656940; doi:10.3389/fnhum.2021.784522)
Supplement: Supplementary file 1 [file Data_Sheet_1.PDF]

Supplementary Material

Material and Methods

Autobiographical Questions

1. Which country were you born in?

- ☐ The Netherlands
- ☐ Germany
- ☐ Belgium
- ☐ Other

2. Which country are you currently living in?

- ☐ The Netherlands
- ☐ Germany
- ☐ Belgium
- ☐ Other

3. Do you have any siblings?

- ☐ No, I do not
- ☐ Yes, only brother(s)
- ☐ Yes, only sister(s)
- ☐ Brother(s) and sister(s)

4. Which colour was your first car?

- ☐ I never owned a car
- ☐ My first car was red
- ☐ My first car was blue
- ☐ It was neither red nor blue

5. What is your current housing situation?

- ☐ Living with partner
- ☐ Living in shared house
- ☐ Living by myself
- ☐ Living with parents

6. Do you have a godchild/godchildren?

- ☐ Yes, only girl(s)
- ☐ Yes, only boy(s)
- ☐ Yes, only boy(s) and girl(s)
- ☐ No, I do not

Motor Imagery Abilities Questionnaire

1. With your dominant hand, please draw a rough sketch of a house.

2. Now imagine drawing the same sketch without actually doing so. Try imagining movements most similar to those used when actually drawing (e.g. wrist and whole hand movements).

3. How vivid was your imagination of drawing the sketch? (Please circle one of the numbers)

0	1	2	3	4
Not very vivid				Very vivid

4. How similar was your imagination of drawing the sketch compared to the actual drawing? (Please circle one of the numbers)

0	1	2	3	4
Not similar at all				Almost identical

5. How easy did you find it to imagine drawing the sketch? (Please circle one of the numbers)

0	1	2	3	4
Not easy at all				Very easy

6. How would you rate your imagination in general? (Please circle one of the numbers)

0	1	2	3	4
Not good<br>At all				Very<br>good

7. How much do you enjoy this task? (Please circle one of the numbers)

0	1	2	3	4
Not at<br>all				Very<br>much

Questionnaire of Strategy and Comfort

1. Please shortly describe what exactly you imagined during the “mental drawing“ task. Draw here what objects/images you drew in your mind.

2. How well did it work in general? Were there any problems? Did you realize any differences between the different trials/runs? Do you have any remarks/suggestions?

3. How comfortable did you feel during the session? (Please circle one number)

1 2 3 4 5 6 7 8 9 10

Not comfortable at all — Very comfortable

4. How comfortable was the cap? (Please circle one number)

1 2 3 4 5 6 7 8 9 10

Not comfortable at all — Very comfortable

5. How tired did you become throughout the session? (Please circle one number)

1 2 3 4 5 6 7 8 9 10

Very tired — Not tired at all

Questionnaire of General Study Impression

Motivation and General Impression

	Strongly disagree	Disagree	Neutral	Agree	Strongly agree
1. I found the study interesting.	☐	☐	☐	☐	☐
2. I enjoyed the task.	☐	☐	☐	☐	☐
3. I was motivated to perform the study.	☐	☐	☐	☐	☐
4. My motivation got lower throughout the study.	☐	☐	☐	☐	☐
5. My motivation got higher throughout the study.	☐	☐	☐	☐	☐
6. I got bored throughout the study.	☐	☐	☐	☐	☐
7. I got tired throughout the study.	☐	☐	☐	☐	☐

Prior Experience

	No	Once	Twice	3-4	>4
8. I have participated in experiments measuring brain-activity (EEG, fMRI, fNIRS, MEG, PET).	☐	☐	☐	☐	☐
9. Have you participated in BCI experiments?	☐	☐	☐	☐	☐
10. Have you participated in neurofeedback experiments?	☐	☐	☐	☐	☐

If you have answered question 9 with **yes** (otherwise skip):

	No	Once	Twice	3-4	>4
11. Have you participated in EEG BCI experiments?	☐	☐	☐	☐	☐
12. Have you participated in fMRI BCI experiments?	☐	☐	☐	☐	☐
13. Have you participated in fNIRS BCI experiments?	☐	☐	☐	☐	☐

If you have answered question 10 with **yes** (otherwise skip):

	No	Once	Twice	3-4	>4
14. Have you participated in EEG neurofeedback experiments.	☐	☐	☐	☐	☐
15. Have you participated in fMRI neurofeedback experiments.	☐	☐	☐	☐	☐
16. Have you participated in fNIRS neurofeedback experiments.	☐	☐	☐	☐	☐

Mental Imagery

	Strongly disagree	Disagree	Neutral	Agree	Strongly agree
17. My imagination of mental drawing was well throughout the whole study.	☐	☐	☐	☐	☐
18. My imagination was similar to real drawing.	☐	☐	☐	☐	☐
19. My imagination got more realistic throughout the study.	☐	☐	☐	☐	☐

	Strongly disagree	Disagree	Neutral	Agree	Strongly agree
20. My imagination got less realistic throughout the study.	☐	☐	☐	☐	☐

21. My imagination of drawing was very vivid.	☐	☐	☐	☐	☐
22. My imagination got more vivid throughout the experiment.	☐	☐	☐	☐	☐
23. My imagination got less vivid throughout the experiment.	☐	☐	☐	☐	☐

Emotions

	Strongly disagree	Disagree	Neutral	Agree	Strongly agree
24. I felt confident using the system.	☐	☐	☐	☐	☐
25. I felt comfortable using the system.	☐	☐	☐	☐	☐
26. I felt anxious using the system.	☐	☐	☐	☐	☐
27. I felt stressed using the system.	☐	☐	☐	☐	☐
28. I felt excited using the system.	☐	☐	☐	☐	☐
29. I felt natural using the system.	☐	☐	☐	☐	☐
30. I am satisfied with my performance.	☐	☐	☐	☐	☐
31. I am satisfied with the performance of the system.	☐	☐	☐	☐	☐
32. I got frustrated throughout the experiment.	☐	☐	☐	☐	☐

Modalities

1. How pleasant did you find the **auditory** guidance? (Please circle one number)

1 2 3 4 5 6 7 8 9 10

Not pleasant at all — Very pleasant

2. How easy did you find the **auditory** guidance? (Please circle one number)

1 2 3 4 5 6 7 8 9 10

Not easy at all — Very easy

3. How pleasant did you find the **visual** guidance? (Please circle one number)

1 2 3 4 5 6 7 8 9 10

Not pleasant at all — Very pleasant

4. How easy did you find the **visual** guidance? (Please circle one number)

1 2 3 4 5 6 7 8 9 10

Not easy at all — Very easy

5. How pleasant did you find the **tactile** guidance? (Please circle one number)

	1	2	3	4	5	6	7	8	9	10
Not pleasant at all										Very pleasant

6. How easy did you find the **tactile** guidance? (Please circle one number)

	1	2	3	4	5	6	7	8	9	10
Not easy at all										Very easy

7. Rate your liking of the three modalities overall (1 = best, 2 = medium, 3 = worst). If you liked two modalities equally, rate them with the same number.

Auditory ☐ Visual ☐ Tactile ☐

8. Please give a brief explanation why.

____

____

Other

9. Was there any question that was ambiguous to you? (Unclear which answer to choose)

☐ yes ☐ no

If so, which one(s) ____

10. Was there any question that elicited strong emotions?

☐ yes ☐ no

If so, which one(s) ____

11. Do you have any other remarks on the study?

Results

Subject-Specific Channel Selection

Based on the localizer data, three channels were selected for each participant in each session. The channel selection for each of the six participants can be gauged in Supplementary Figure 1. The EEG coordinates of these channels are displayed in Supplementary Table 1. The channel selection frequency across the six participants is partly depicted in Supplementary Figure 2. All absolute frequencies can be read from Supplementary Table 2.

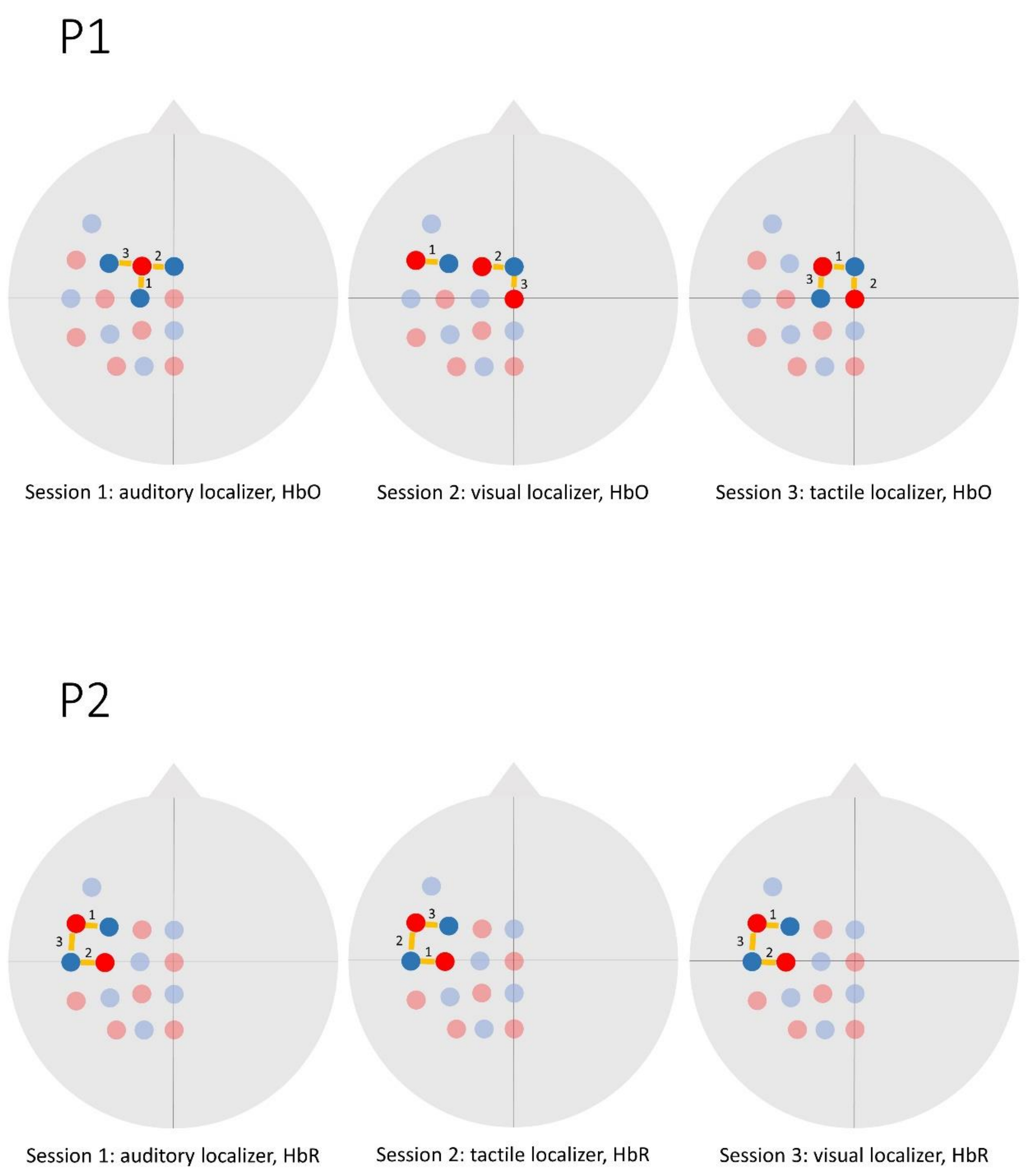

P3

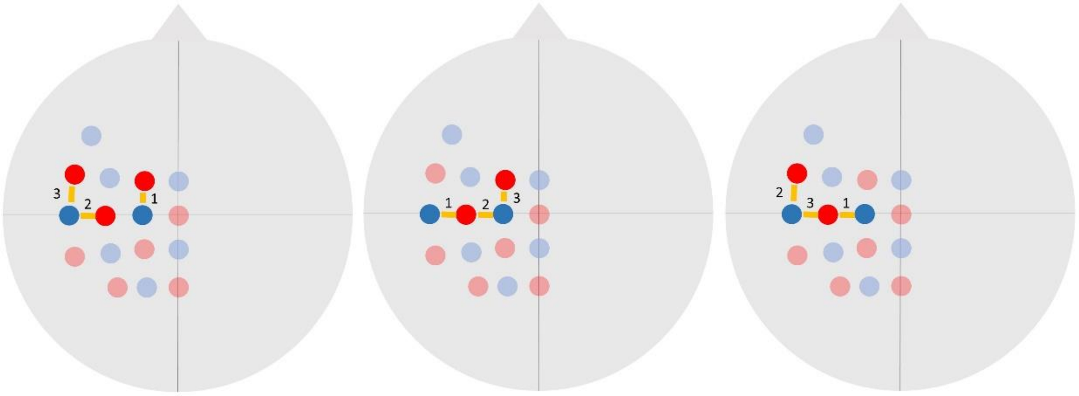

Session 1: visual localizer, HbO   Session 2: tactile localizer, HbO   Session 3: auditory localizer, HbO

P4

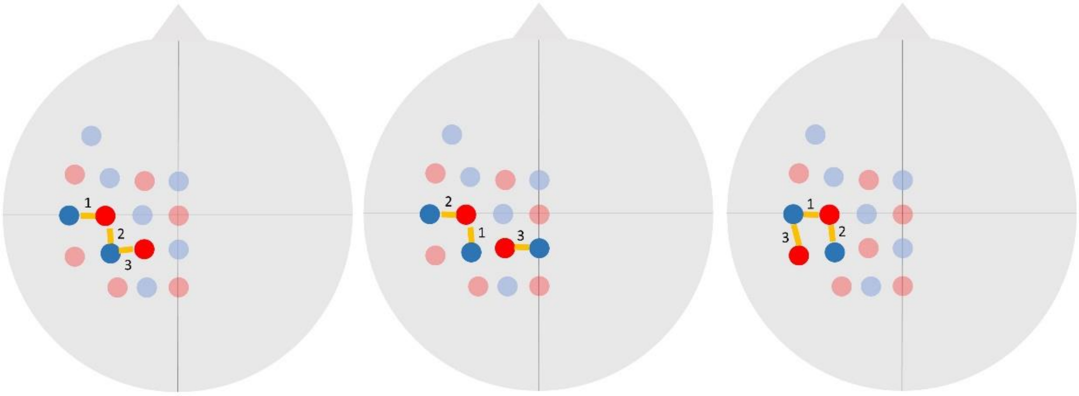

Session 1: tactile localizer, HbR   Session 2: auditory localizer, HbR   Session 1: visual localizer, HbO

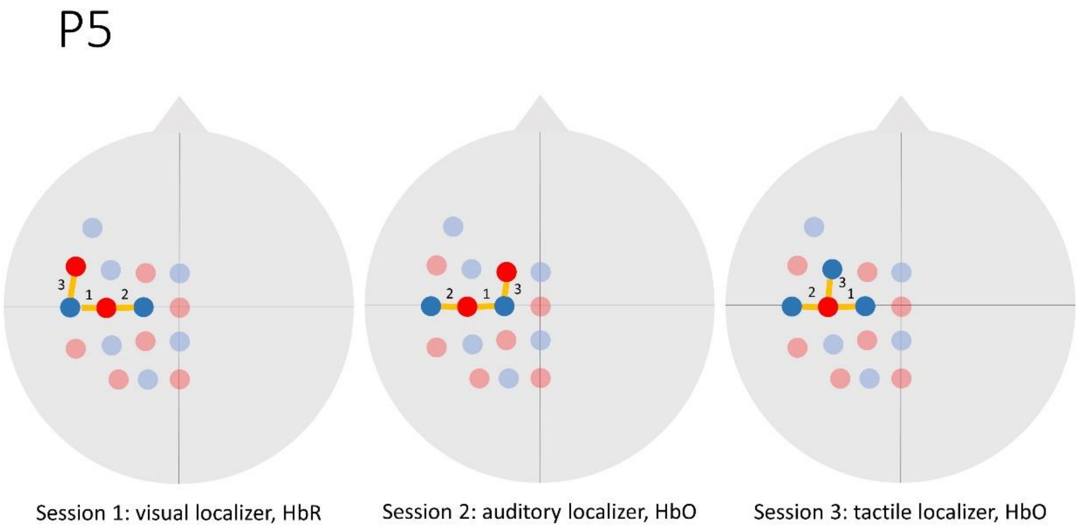

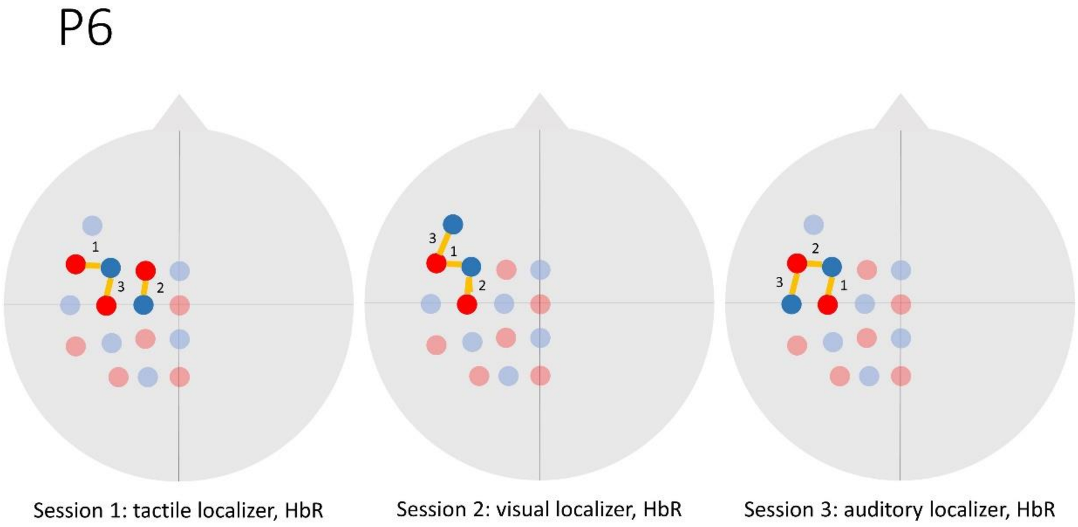

SUPPLEMENTARY FIGURE 1| Individual Participant's Channel Selection. For each participant the most informative channels are displayed for each fNIRS session. The yellow lines between a source (red) and detector (blue) optode indicate the channel-by-chromophore that were chosen as the most informative channel (1), the 2nd most informative channel (2) and the 3rd most informative channel (C3). *Abbreviations:* HbO, oxygenated hemoglobin; HbR, deoxygenated hemoglobin.

PARTICIPANT	SESSION	CHROMOPHORE	COI1	COI2	COI3
**P1**	Session 1	HbO	FC1-C1	FC1-FCZ	FC1-FC3
	Session 2		FC5-FC3	FC1-FCZ	CZ-FCZ
	Session 3		FC1-FCZ	CZ-FCZ	FC1-C1
**P2**	Session 1	HbR	FC5-FC3	C3-C5	FC5-C5
	Session 2		C3-C5	FC5-C5	FC5-FC3
	Session 3		FC5-FC3	C3-C5	FC5-C5
**P3**	Session 1	HbO	FC1-C1	C3-C5	FC5-C5
	Session 2		C3-C5	C3-C1	FC1-C1
	Session 3		C3-C1	FC5-C5	C3-C5
**P4**	Session 1	HbR	C3-C5	C3-CP3	CP1-CP3
	Session 2	HbR	C3-CP3	C3-C5	CP1-Cpz
	Session 3	HbO	C3-C5	C3-CP3	CP5-C5
**P5**	Session 1	HbR	C3-C5	C3-C1	FC5-C5
	Session 2	HbO	C3-C1	C3-C5	FC1-C1
	Session 3	HbO	C3-C1	C3-C5	C3-FC3
**P6**	Session 1	HbR	FC5-FC3	FC1-C1	C3-FC3
	Session 2		FC5-FC3	C3-FC3	FC5-F5
	Session 3		C3-FC3	FC5-FC3	FC5-C5

SUPPLEMENTARY TABLE 1| Individual Channel × Chromophore Selection. In each session, the most promising channel-by-chromophore combination was selected for every participant. The last three columns show the channels-of-interest (COI). *Abbreviations:* HbO, oxygenated hemoglobin; HbR, deoxygenated hemoglobin; COI1, most informative channel; COI2, 2nd most informative channel; COI3, 3rd most informative channel.

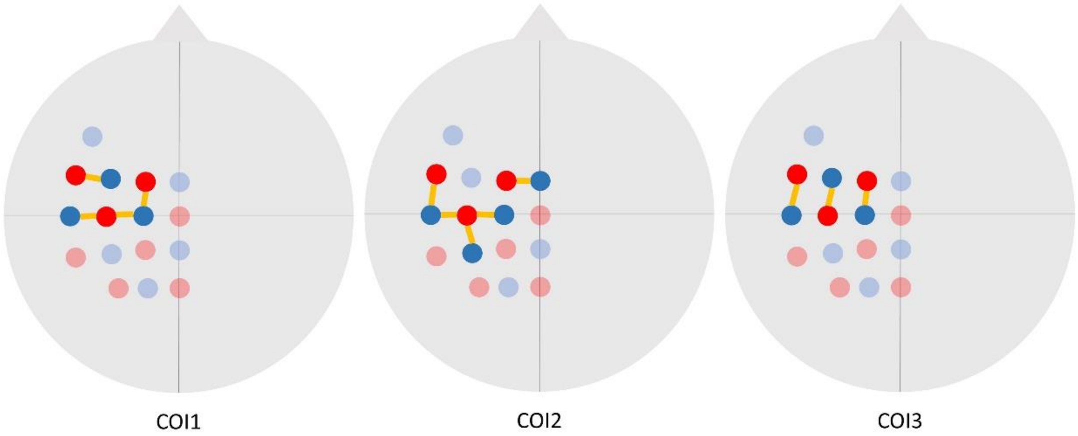

SUPPLEMENTARY FIGURE 2 | Channel Selection Frequency. Channel selection frequency (n=18; 6 participants x 3 fNIRS session) of the most informative channel (COI1), the 2nd most informative channel (COI2) and the 3rd most informative channel (COI3). The yellow lines between a source (red) and detector (blue) optode indicate informative channels that were chosen more than once as informative out of 18 cases.

FNIRS CHANNEL	FREQUENCY COI1	FREQUENCY COI2	FREQUENCY COI3
**FC5-F5**	0	0	1
**FC5-C5**	0	2	5
**FC5-FC3**	5	1	1
**C3-C5**	5	6	1
**C3-FC3**	1	1	2
**C3-CP3**	1	2	0
**C3-C1**	3	2	0
**CP5-C5**	0	0	1
**CP5-CP3**	0	0	0
**P3-CP3**	0	0	0
**P3-P1**	0	0	0
**FC1-FC3**	0	0	1
**FC1-FCZ**	1	2	0
**FC1-C1**	2	1	3
**CZ-FCZ**	0	1	1
**CZ-C1**	0	0	0
**CZ-CPZ**	0	0	0
**CP1-CP3**	0	0	1
**CP1-C1**	0	0	0
**CP1-CPZ**	0	0	1
**CP1-P1**	0	0	0
**PZ-CPZ**	0	0	0
**PZ-P1**	0	0	0
**SUM**	18	18	18

SUPPLEMENTARY TABLE 2 | Absolute Channel Selection Frequency. The fNIRS channels identified by their EEG coordinates (left column) and the absolute selection frequency of the most informative channel (COI1), the 2nd most informative channel (COI2) and the 3rd most informative channel (COI3). The sum of absolute frequency is 18 (6 participants and 3 fNIRS sessions).

BCI user experience

Experienced Comfort and Tiredness across fNIRS Sessions

General comfortability, cap comfortability and tiredness scores remained relatively stable over the three fNIRS sessions (see Supplementary Figure 3).

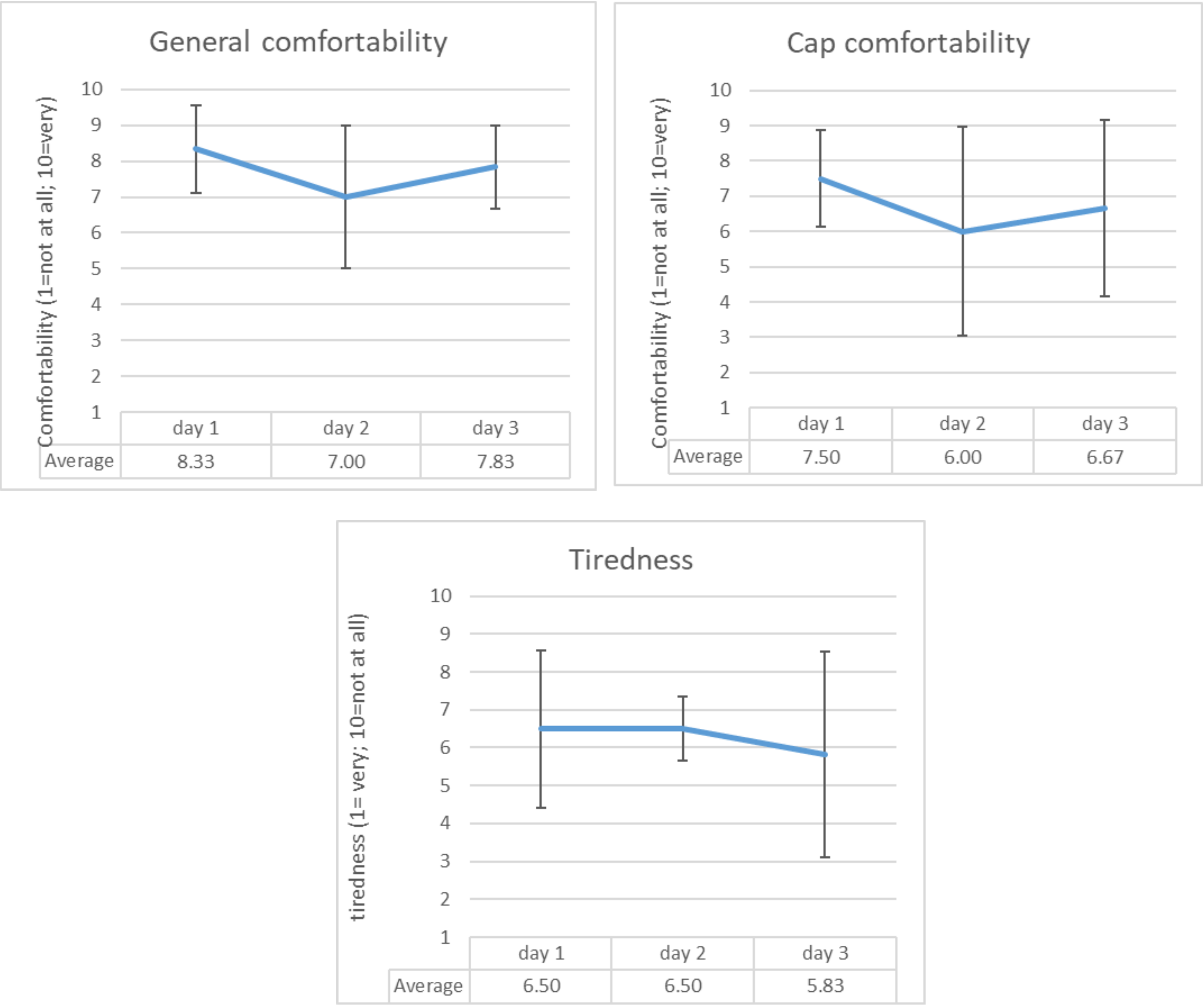

SUPPLEMENTARY FIGURE 3 | Mean Comfortability, Cap Comfortability and Fatigue across fNIRS sessions. Participants rated each aspect on a 10-point Likert scale (1 indicating 'uncomfortable/very tired' and 10 indicating 'very comfortable/not tired at all'). The error bars depict the standard deviation of the group mean. Note that all ratings remained relatively stable across the sessions.

Motion Imagery Questionnaire

The self-reported motor imagery ability scores (0-20) correlated significantly with the multi-trial decoding accuracies ($r(4)= 0.95$; $p < 0.01$), but not with the single-trial decoding accuracies ($r(4)= 0.73$; $p = 0.10$). Participants that rated their motor imagery ability as high, tended to have a high multi- trial decoding accuracy (see Supplementary Figure 4).

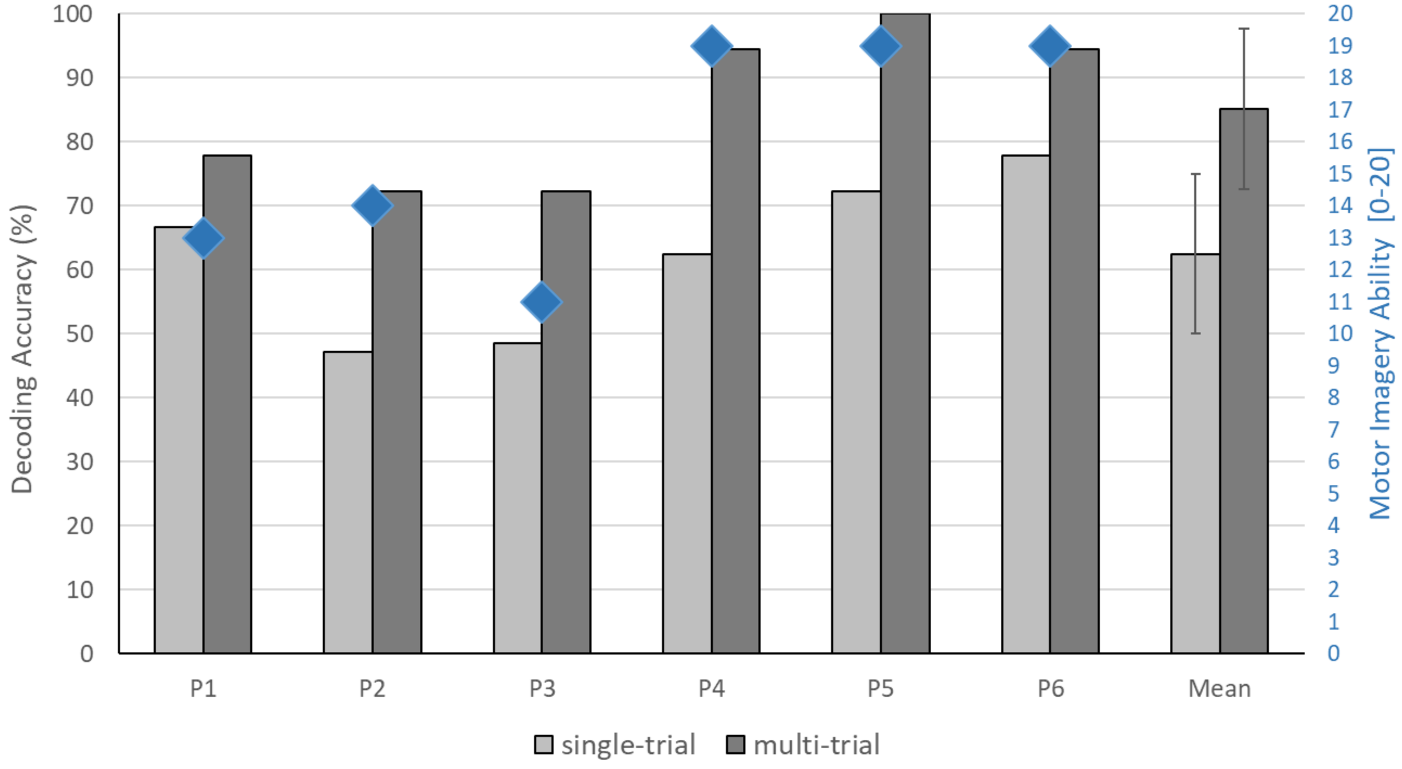

SUPPLEMENTARY FIGURE 4 | Individual and Sample Mean Decoding Accuracies and Motor Imagery Ability Score. The single- (light grey) and multi-trial decoding accuracies can be interpreted on the primary axis (left). Error bars depict the standard deviation of the group mean. Motor imagery ability scores are depicted with blue diamonds and can be interpreted using the secondary axis (right). Note the association between the participants' self-reported motor imagery ability and their communication success.
